# Relationships of Perfluorooctanoate and Perfluorooctane Sulfonate Serum Concentrations between Mother–Child Pairs in a Population with Perfluorooctanoate Exposure from Drinking Water

**DOI:** 10.1289/ehp.1104538

**Published:** 2012-01-23

**Authors:** Debapriya Mondal, Maria-Jose Lopez-Espinosa, Ben Armstrong, Cheryl R. Stein, Tony Fletcher

**Affiliations:** 1 London School of Hygiene and Tropical Medicine, London, United Kingdom; 2 Department of Preventive Medicine, Mount Sinai School of Medicine, New York, New York, USA

**Keywords:** mother–child pairs, drinking water, *in utero* exposure, lactation, Mid-Ohio Valley, PFOA, PFOS, serum concentration

## Abstract

Background: There are limited data on the associations between maternal or newborn and child exposure to perfluoroalkyl acids (PFAAs), including perfluorooctanoate (PFOA) and perfluorooctane sulfonate (PFOS). This study provides an opportunity to assess the association between PFAA concentrations in mother–child pairs in a population exposed to PFOA via drinking water.

Objectives: We aimed to determine the relationship between mother–child PFAA serum concentrations and to examine how the child:mother ratio varies with child’s age, child’s sex, drinking-water PFOA concentration, reported bottled water use, and mother’s breast-feeding intention.

Methods: We studied 4,943 mother–child pairs (children, 1–19 years of age). The child:mother PFAA ratio was stratified by possible determinants. Results are summarized as geometric mean ratios and correlation coefficients between mother–child pairs, overall and within strata.

Results: Child and mother PFOA and PFOS concentrations were correlated (*r* = 0.82 and 0.26, respectively). Up to about 12 years of age, children had higher serum PFOA concentrations than did their mothers. The highest child:mother PFOA ratio was found among children ≤ 5 years (44% higher than their mothers), which we attribute to *in utero* exposure and to exposure via breast milk and drinking water. Higher PFOS concentrations in children persisted until at least 19 years of age (42% higher than their mothers). Boys > 5 years of age had significantly higher PFOA and PFOS child:mother ratios than did girls.

Conclusion: Concentrations of both PFOA and PFOS tended to be higher in children than in their mothers. This difference persisted until they were about 12 years of age for PFOA and at least 19 years of age for PFOS.

Perfluoroalkyl acids (PFAAs), including perfluorooctanoate (PFOA) and perfluorooctane sulfonate (PFOS), are persistent environmental pollutants that have been detected worldwide in both wildlife and humans, with higher exposure closer to urbanized and industrialized regions (Houde et al. 2011). PFAAs have numerous industrial uses, ranging from the manufacture of fluoropolymers, mainly used for nonstick cookware and breathable yet waterproof fabrics; coatings for carpets, paper, and textile; food-packing materials; electronic and photographic devices; surfactants in diverse cleaning agents; cosmetics; and fire-fighting foams (Organisation for Economic Co-operation and Development 2005). Direct industrial emissions are estimated to be the major source of these compounds in the environment. PFAAs can also come from the breakdown of PFAA-containing products and from the “precursor” compounds, such as fluorinated telomers (Vestergren and Cousins 2009), used in diverse commercial and industrial applications, including paints, coatings, polymers, adhesives, waxes, polishes, electronics, and caulks (Kissa 2001). The ubiquitous presence and long half-lives of PFOA and PFOS have led to both voluntary and regulatory control measures, leading to manufacturing phase-out (Sundstrom et al. 2011).

In the general population, food intake is the major exposure pathway to PFOA and PFOS, whereas drinking water is the dominant exposure route in populations living near contaminated water sources (Vestergren and Cousins 2009). The median concentrations in non-occupationally exposed adult populations ranged from 1.6 to 11.6 ng/mL for PFOA and 3.3 to 55.8 ng/mL for PFOS [reviewed by Fromme et al. (2009)]. Median PFOA and PFOS concentrations were found to be 5.1 and 36.7 ng/mL, respectively, in 2- to 12-year-old children from the United States, 4.9 and 4.3 ng/mL in 5- to 6-year-old children from Europe, and 3.6 and 7.3 ng/mL in newborns from Hungary [reviewed by Fromme et al. (2009)]. Another study by Zhang et al. (2010) reported median PFOA and PFOS ranges from 1.0 to 2.4 ng/mL and 2.5 to 5.6 ng/mL, respectively, in children 0–18 years of age from China.

Unlike adult exposure, which derives primarily from food and water, gestation and breast-feeding are considered important routes of infant exposure (Fromme et al. 2010; Haug et al. 2011; Tao et al. 2008; Thomsen et al. 2010). Prenatal transfer of PFOA and PFOS from mothers to newborns has been demonstrated by detection of these contaminants in maternal and umbilical cord blood samples (Apelberg et al. 2007; Fei et al. 2007; Fromme et al. 2010; Hanssen et al. 2010; Inoue et al. 2004; Kim et al. 2011; Midasch et al. 2007; Monroy et al. 2008). Most studies (Fei et al. 2007; Fromme et al. 2010; Hanssen et al. 2010; Inoue et al. 2004; Kim et al. 2011; Monroy et al. 2008; Needham et al. 2011) reported higher PFOA and PFOS concentrations in maternal blood than in cord blood (cord:maternal concentration ratios for paired samples = 0.67–0.87 for PFOA and 0.28–0.56 for PFOS). Conversely, one study (Midasch et al. 2007) found higher cord blood than maternal concentrations (median cord:maternal PFOA ratio = 1.3). The transfer of maternal PFOA and PFOS to the newborns has also been demonstrated by detection of these contaminants in paired breast milk and infant blood samples (Fromme et al. 2010). In different populations, average breast milk PFAA concentrations ranged from 2.5% to 3.8% of the mothers’ serum concentration for PFOA (Haug et al. 2011; Kim et al. 2011) and from 0.9% to 1.4% for PFOS (Fromme et al. 2010; Haug et al. 2011; Karrman et al. 2007; Kim et al. 2011).

Exposure of the general U.S. population to PFOA and PFOS is widespread (Calafat et al. 2007). PFOA has been used in the manufacture of fluoropolymers at a chemical plant in the Mid-Ohio Valley near Parkersburg, West Virginia (USA), since 1951. In 2001, a group of residents from the West Virginia and Ohio communities surrounding the plant filed a class action lawsuit alleging health damage due to contamination of human drinking water supplies with PFOA (Frisbee et al. 2009). The settlement of this class action lawsuit led to a baseline survey, called the C8 Health Project, conducted in 2005–2006 that gathered data from > 69,000 people who lived in the six contaminated water districts surrounding the plant (Frisbee et al. 2009), including demographic and health questionnaires and measurement of 10 PFAAs in serum. Groundwater contamination from the Ohio River and air deposition are believed to be the primary exposure routes for this population (Shin et al. 2011). Median PFOA and PFOS serum concentrations in the population were 28 and 20 ng/mL, respectively (Frisbee et al. 2009), compared with 4.2 and 17.5 ng/mL in the 2005–2006 U.S. National Health and Nutrition Examination Survey population (Kato et al. 2011), indicating that this community was exposed to PFOA above the background levels.

Although entire families may have enrolled in the C8 Health Project, eligibility was determined on an individual basis, and study records were maintained individually; that is, families within the C8 Health Project population were not identified as such. The aim of the present investigation was to match children (1–19 years of age) to their mothers among survey participants and then to describe the relationship between child and mother PFOA and PFOS serum concentrations measured at C8 Health Project survey enrollment. We also examined the impact of child’s age, child’s sex, drinking-water PFOA concentration, bottled water use, and mother’s breast-feeding intention on the child:mother PFOA and PFOS ratios.

## Methods

*Study population.* The C8 Health Project enrolled subjects between August 2005 and July 2006. All participants gave written informed consent before inclusion. The London School of Hygiene and Tropical Medicine Ethics Committee approved this study. Enrollment criteria and consent procedures are described in a previous publication (Frisbee et al. 2009). Briefly, subjects were eligible if they could document drinking-water consumption for at least 1 year before December 2004 from *a*) a public source from any of the six PFOA-contaminated water districts [City of Belpre, Ohio (WD-1); Tuppers Plains Chester Water District of Ohio (WD-2); Little Hocking Water Association of Ohio (WD-3); Lubeck Public Service District of West Virginia (WD-4); Mason County Public Service District of West Virginia (WD-5); or Village of Pomeroy, Ohio (WD-6)] or *b*) a private well known to be contaminated with PFOA. Individuals who had either worked or attended school in a contaminated water district for at least 1 year were also eligible. The C8 Health Project collected data on 69,030 people. Based on population estimates for census block groups in 2005, the participation rate of people still living in the exposed water districts at survey was estimated as 80% (Frisbee et al. 2009). Within the C8 Health Project population, 48,880 (69%) further consented to provide their detailed contact information (full name, date of birth, and residential address) to allow participation in the further studies. The present analysis is restricted to this subset of identified participants. A total of 8,893 of the 48,880 participants were children < 20 years of age at enrollment, and these children constitute our “child file” for matching. Of these children, 6,519 (73%) had a parent’s name contained within the child’s survey record to establish who completed the questionnaire for the child (these forms were assembled as a “guardian file”). Among the adult women participants, 17,543 (69%) reported a pregnancy history (17,063 reported live births representing 39,289 child births), including the month and year of the child’s birth and child’s sex. These women constitute the “mother file” for matching.

*Matching of children to mothers.* We used two methods for matching the biological children to their mothers. First, we used “identifier matching,” where the mother and child last names, street address, ZIP code, and phone number and child’s sex and date of birth (year and month) were matched between the child and mother files. Second, we used “guardian matching” to try to confirm the mother–child pairs if the named guardian was identified as the mother. Lack of confirmation in the guardian file may mean that the child was not in the guardian file or that someone other than the child’s mother completed the survey on the child’s behalf. Matches made through identifier matching that were refuted by the guardian file were not included in the analysis. Finally, we classified the matched pairs as perfect, excellent, good, or probable [see Supplemental Material, Table 1 (http://dx.doi.org/10.1289/ehp.1104538)] based on the number of elements that matched exactly, with perfect being the best.

**Table 1:** Summary PFAA statistics (ng/mL) for the matched mother–child pairs (n = 4,943), Mid-Ohio Valley, 2005–2006.

PFAA	AM ± SD	GM (GSD)	Minimum	P10	P50	P90	Maximum
PFOA														
Child		68.4 ± 111		31.2 (3.25)		0.70		8.00		26.1		201		1,283
Mother		73.6 ± 218		27.2 (3.62)		0.25		6.30		22.3		182		8,163
Child:mother ratio		1.52 ± 1.80		1.15 (2.05)		0.01		0.48		1.13		2.78		49.2
PFOS														
Child		22.0 ± 12.1		19.2 (1.76)		0.25		10.2		19.3		36.8		152
Mother		16.3 ± 10.9		13.4 (1.97)		0.25		6.40		14.2		28.2		225
Child:mother ratio		2.11 ± 5.50		1.43 (2.10)		0.009		0.62		1.37		3.42		208
Abbreviations: AM, arithmetic mean; P, percentile.														

*Serum PFOA and PFOS determination.* The method used for measurement of serum PFOA and PFOS is described elsewhere (Frisbee et al. 2009). Briefly, blood samples were obtained and processed at individual data collection sites after the enrollment of the study participants during 2005–2006, and serum concentrations of PFOA and PFOS were determined using liquid chromatography separation with detection by tandem mass spectrometry. Estimates of precision for PFOA were within ± 10% for multiple replicates over the range of 0.50–40 ng/mL, with a more precise relative precision measure of approximately 1% for highly fortified (10,000 ng/mL) samples. Relative precision estimates for PFOS were similar. The detection limit (LOD) for both PFOA and PFOS was 0.50 ng/mL, and observations below LOD were assigned a value of 0.25 ng/mL.

*Determinants of the child:mother ratio.* We investigated how the child:mother PFOA ratio varied by the following parameters: *a*) child’s age at survey [≤ 5, 6–10, > 10 years, or 1-year increment; see Supplemental Material, Tables 5 and 6 (http://dx.doi.org/10.1289/ehp.1104538)], *b*) child’s sex, *c*) reported use of bottled water for drinking (categorized as by child not mother, by mother not child, or by neither), *d*) level of potential exposure based on which water district the child and mother were living in (restricted to those matched pairs where the child and mother lived in the same water district from the time of the child’s birth to the time of the survey), and *e*) mother’s intention to breast-feed as recorded on the West Virginia Birth Score Developmental Risk Screen conducted after delivery (available for a subset only). Intention to breast-feed was classified as exclusive breast-feeding versus breast- and/or bottle feeding. We investigated modification of the child:mother PFOS ratio by child’s age at survey, child’s sex, and mother’s intention to breast-feed.

*Statistical analysis.* Given positively skewed distributions of serum PFAA concentrations and the child:mother ratios, we summarized the center of their distributions as geometric means (GMs). We described the strength of the association between child and mother serum PFOA and PFOS concentrations using both scatter plots and Spearman correlation coefficients.

After preliminary regression analyses, we summarized each mother–child measurement pair as the child:mother concentration ratio. We described the predictors of the child:mother PFAA ratios by tabulating the GM of the ratio by each putative predictor. Confidence intervals (CIs) for the GMs of group-specific ratios and significance tests for group differences (Wald tests) were obtained by regressions with the log child:mother ratio as the outcome and categorical explanatory variables (equivalent to analysis of variance). Statistical significance was taken as *p* < 0.05, two-sided.

We used the statistical software package STATA for all statistical analyses (version 11; StataCorp, College Station, TX, USA).

## Results

We matched 6,301 of the 8,893 children (71%) to their mothers (82% for children ≤ 5 years of age); of these, 5,589 (62%, increasing to 73% for children ≤ 5 years) matched with a high degree of confidence (perfect or excellent). Supplemental Material, Table 2 (http://dx.doi.org/10.1289/ehp.1104538), shows the frequency of successful matches stratified by the age of the child and the certainty of the matching (perfect, excellent, good, and probable). A total of 4,943 of 5,589 pairs had measured serum PFOA and PFOS concentrations for both the mother and child and were considered for further analyses. The Spearman correlation coefficients of PFOA for matched pairs classified as perfect (70% of total matches) and excellent (18% of total matches) were 0.82 and 0.80, respectively. Respective data for PFOS was 0.27 for both matches (see Supplemental Material, Table 3).

**Table 2:** GMs of child:mother PFOA and PFOS ratios and Spearman correlation coefficients (ρ) stratified by child’s age groups, Mid-Ohio Valley, 2005–2006.

All					Stablea				
Child’s age (years)	n	Ratio (95% CI)	ρ	n	Ratio (95% CI)	ρ
PFOA												
All (1–19)		4,943		1.15 (1.12, 1.17)		0.82		1,763		1.10 (1.07, 1.14)		0.86
≤ 5		485		1.44 (1.35, 1.53)		0.82		266		1.37 (1.27, 1.49)		0.86
6–10		1,334		1.31 (1.26, 1.36)		0.84		536		1.26 (1.20, 1.34)		0.85
> 10		3,124		1.04 (1.02, 1.07)		0.82		961		0.96 (0.92, 1.01)		0.87
PFOS												
All (1–19)		4,943		1.42 (1.40, 1.46)		0.26		1,763		1.38 (1.33, 1.43)		0.23
≤ 5		485		1.34 (1.25, 1.43)		0.27		266		1.27 (1.15, 1.39)		0.27
6–10		1,334		1.63 (1.57, 1.69)		0.25		536		1.59 (1.49, 1.69)		0.22
> 10		3,124		1.36 (1.33, 1.40)		0.27		961		1.31 (1.25, 1.37)		0.24
aThe mother and child remained in the same water district (the six contaminated districts, WD-1 through WD-6) from the child’s birth up to the survey.												

**Table 3:** GMs of child:mother PFOA and PFOS ratios stratified by child’s sex and age groups, Mid-Ohio Valley, 2005–2006.

All			Girls			Boys		
Child’s age (years)	n	Ratio (95% CI)	n	Ratio (95% CI)	n	Ratio (95% CI)
PFOA												
All (1–19)		4,943		1.15 (1.12, 1.17)		2,464		1.05 (1.02, 1.08)		2,479		1.25 (1.21, 1.28)
≤ 5		485		1.44 (1.35, 1.53)		251		1.43 (1.31, 1.56)		234		1.45 (1.33, 1.58)
6–10		1,334		1.31 (1.26, 1.36)		673		1.23 (1.17, 1.30)		661		1.40 (1.33, 1.46)
> 10		3,124		1.04 (1.02, 1.07)		1,540		0.94 (0.90, 0.97)		1,584		1.16 (1.12, 1.20)
PFOS												
All (1–19)		4,943		1.42 (1.40, 1.46)		2,464		1.35 (1.31, 1.39)		2,479		1.51 (1.47, 1.56)
≤ 5		485		1.34 (1.25–1.43)		251		1.31 (1.19, 1.44)		234		1.37 (1.24, 1.51)
6–10		1,334		1.63 (1.57–1.69)		673		1.56 (1.48, 1.65)		661		1.70 (1.61, 1.80)
> 10		3,124		1.36 (1.33–1.40)		1,540		1.27 (1.22, 1.31)		1,584		1.46 (1.41, 1.52)

**Figure 1:**
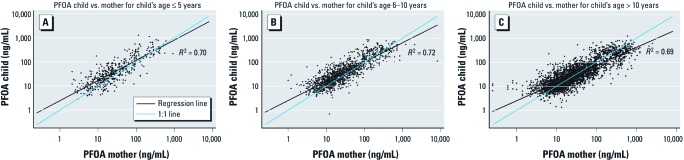
Child versus maternal PFOA (log scale) by child’s age group, Mid-Ohio Valley, 2005–2006 (*n* = 4,943): (*A*) children ≤ 5 years of age, (*B*) children 6–10 years of age, (*C*) children > 10 years of age.

**Figure 2:**
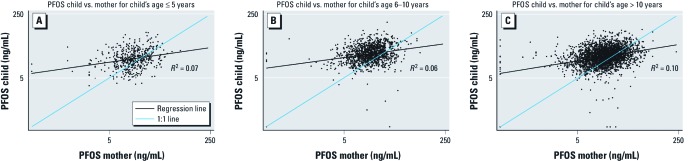
Child versus maternal PFOS (log scale) by child’s age group, Mid-Ohio Valley, 2005–2006 (*n* = 4,943): (*A*) children ≤ 5 years of age, (*B*) children 6–10 years of age, (*C*) children > 10 years of age.

Table 1 presents the summary statistics for the matched pairs, and Figures 1 and 2 show scatterplots of child versus mother PFOA and PFOS concentrations, respectively, by age group. The GMs of PFOA and PFOS serum concentrations for children were higher than those of their mothers. The geometric standard deviation (GSD) overall for PFOA concentrations is about twice that for PFOS, whereas the GSDs for the child:mother ratios are virtually identical for PFOA and PFOS. The higher correlation coefficients for PFOA than for PFOS partly reflect this difference, as is evident in comparing Figures 1 and 2. The regression slopes shown on the figures [for details, see Supplemental Material, Table 4 (http://dx.doi.org/10.1289/ehp.1104538)] are below the 1:1 lines (proportionality), and this is expected even where true slopes are equal to 1 if both the *x* and *y* variables are subject to error, such as anticipated here because of short-term within-person variation in mothers’ PFAA concentrations (Armstrong 1998).

**Table 4:** GMs of child:mother PFOA ratios stratified by exposure status and child’s age, Mid-Ohio Valley, 2005–2006.

Child’s age (years)		All			High exposurea			Medium exposureb			Low exposurec		
		n	Ratio (95% CI)	n	Ratio (95% CI)	n	Ratio (95% CI)	n	Ratio (95% CI)
≤ 5		266		1.37 (1.27, 1.49)		70		1.57 (1.33, 1.85)		141		1.41 (1.26, 1.58)		55		1.07 (0.93, 1.23)
6–10		536		1.26 (1.20, 1.34)		121		1.24 (1.08, 1.42)		271		1.36 (1.26, 1.46)		144		1.12 (1.01, 1.25)
> 10		961		0.96 (0.92, 1.00)		225		0.92 (0.83, 1.02)		463		0.97 (0.91, 1.03)		273		1.00 (0.93, 1.07)
Respective maternal PFOA serum levels are presented in Supplemental Material, Table 7 (http://dx.doi.org/10.1289/ehp.1104538). aMother and child living in WD-3. bMother and child living in WD-1, WD-2, or WD-4. cMother and child living in WD-5 or WD-6.																

**Table 5:** GMs of child:mother PFOA and PFOS ratios (95% CIs) stratified by mother’s intention to breast-feed, for children ≤ 3 years of age and stable since birth (n = 35).

Mother’s feeding intentiona	n	PFOA	PFOS
Exclusive breast-feeding		20		1.83 (1.36, 2.45)		1.35 (0.90, 2.01)
Breast- and/or bottle feeding		15		1.14 (0.66, 1.97)		1.12 (0.61, 2.03)
aIntention was classified as exclusive breast-feeding versus breast- and/or bottle feeding as recorded on West Virginia Birth Score Developmental Risk Screen conducted after delivery.						

Tables 2 and 3 show the GMs of child:mother PFOA and PFOS ratios and the Spearman correlation coefficients stratified by child’s age group and sex, respectively (*n* = 4,943). The child:mother PFOA ratio fell with child age [Table 2; see also Supplemental Material, Table 5 (http://dx.doi.org/10.1289/ehp.1104538)], and for children > 12 years of age the ratio was close to 1.0. In contrast, the child:mother PFOS ratio remained > 1.0 and did not follow a trend with increasing age of the child (Table 2; see also Supplemental Material, Table 6). We also found a sex difference in the child:mother ratios (Table 3), which were higher for boys than for girls (statistically significant for children > 5 years).

A small portion of mothers (6.6%) and children (5.8%) reported using bottled water. The GMs of the child:mother PFOA ratio did not vary by reported bottled water use. The GM ratio of bottled water use by child and not mother was 1.17 (*n* = 66); mother and not child, 1.19 (*n* = 105); and neither, 1.14 (*n* = 4,551).

Table 2 also shows the GMs of child:mother PFOA and PFOS ratios and the Spearman correlation coefficients for the 1,763 matched pairs who lived in the same water district (WD-1 through WD-6) from the child’s birth up to the time of the survey. The PFOA and PFOS ratios for these subgroups (labeled “stable”) are slightly lower than those for the whole population. We considered only this stable matched pair subgroup for further analyses. Supplemental Material, Table 7 (http://dx.doi.org/10.1289/ehp.1104538), presents the GMs of maternal serum PFOA concentration for these 1,763 mother–child pairs classified into three exposure groups (high, medium, and low) according to water district. PFOS concentrations do not vary between these water district classifications (data not shown).

**Figure 3:**
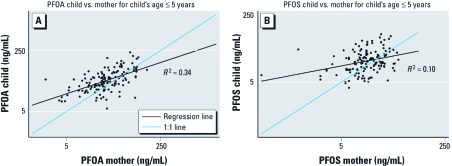
Child versus maternal log-scale PFOA (*A*) and PFOS (*B*) for children ≤ 5 years of age in medium-exposure water districts (WD-1, WD-2, and WD-4), Mid-Ohio Valley, 2005–2006.

Table 4 shows the GMs of child:mother PFOA ratios cross-classified by child age groups and PFOA exposure groups. We observed that the high ratio for children ≤ 5 years of age is more apparent in the high- and medium-exposure areas. Figure 3 shows scatterplots of child versus maternal PFOA and PFOS concentrations for children ≤ 5 years of age living in medium-exposure water districts. In this exposure group, the distributions of serum concentrations of PFOA and PFOS are more similar than in the overall population (which shows a much wider range of PFOA concentrations). In those pairs with children ≤ 5 years, the GMs (GSDs) for the mothers are 25.8 (2.2) ng/mL for PFOA and 12.7 (2.0) ng/mL for PFOS (data not shown). The Spearman correlation for PFOA (0.60) is higher than that for PFOS (0.32) even within this subgroup, perhaps reflecting exposure sources being more heterogeneous for PFOS between young children and their mothers.

Table 5 shows the GMs of the child:mother PFOA and PFOS ratios for children ≤ 3 years of age for whom we have data on the mother’s breast-feeding intention (*n* = 35). There is a suggestion that for PFOA, and to a lesser extent for PFOS, the ratio is higher for the children with mothers who intended to exclusively breast-feed than for mothers with breast- and/or bottle-feeding intentions, although numbers are small, and in neither case was the difference statistically significant.

## Discussion

In the present study, although the original data were not grouped in families, we succeeded in matching 71% of children < 20 years of age at survey to their mothers. For the matches with a high degree of confidence (perfect and excellent, *n* = 4,943) we determined the relationship between child’s and mother’s PFOA and PFOS concentrations and how it varied with child’s age, child’s sex, and bottled water use. Further, for those children who remained at the same address from birth to sampling date (*n* = 1,763), we quantified the relationship between the child’s and mother’s PFOA and PFOS concentrations and how it varied with child’s age, extent of PFOA exposure via drinking water, and the mother’s breast-feeding intention. To our best knowledge, this is the first study looking into the relationship between PFOA and PFOS serum concentrations of both child and mother in paired samples over a wide range of the child’s age (1–19 years) and further looking into the dependence of the relationship on other factors.

Overall, we found a higher correlation between child and mother concentrations for PFOA than for PFOS. The higher correlation could be explained by contaminated drinking water being the major route of exposure for PFOA in this population. Furthermore, drinking-water sources may be more highly correlated within families than are the multiple sources relevant to PFOS uptake, such as food, ingestion of house dust, and inhalation of indoor air (Haug et al. 2009). Even within water districts, the correlations were higher for PFOA, although less than the overall PFOA correlation. The lower correlation for PFOS than for PFOA suggests that the more diverse routes of exposure to PFOS lead to more independence in the intake of PFOS between mother and child. For very young children, the child:mother ratio was higher and the correlation stronger for PFOA than for PFOS, suggesting that transplacental and lactational exposure is more important for PFOA than for PFOS. Our results of a higher child-to-mother correlation for PFOA than for PFOS are in accordance with previous studies (Fei et al. 2007; Fromme et al. 2010; Kim et al. 2011; Needham et al. 2011) examining PFAA correlations between cord and maternal blood samples.

We found a median child:mother PFOA ratio of 1.45 in children 2 years of age [see Supplemental Material, Table 5 (http://dx.doi.org/10.1289/ehp.1104538)], which is much lower than that observed in the study by Fromme et al. (2010): 4.60 in newborns 6 months of age and 3.06 in children 19 months of age. The respective PFOS ratio of 1.03 from Fromme et al. (2010) is comparable to our ratio of 0.98 (see Supplemental Material, Table 6). However, the overall exposure to PFOA as measured in mother’s serum (median of 2.4 vs. 22.3 ng/mL in the present study) and PFOS (3.2 vs. 14.2 ng/mL) is much lower than in our study population. From the reported data by Holzer et al. (2008), we calculated ratios of unpaired children to mothers as 0.98 for PFOA (mean PFOA in mothers, 23.4 ng/mL) and 0.85 for PFOS (mean PFOS in mothers, 5.8 ng/mL) in children with an average age of 5 years, which are lower than the ratios in the present study (1.33 and 1.55) (see Supplemental Material, Table 5 for PFOA and Table 6 for PFOS).

For both PFOA and PFOS, the child:mother ratio was similar by child’s sex up to 5 years of age but was significantly different between girls and boys for children > 5 years of age, with higher ratios for boys than for girls. This could be attributable to differences in water consumption postweaning or biological differences between boys and girls.

The higher concentration of both PFOA and PFOS in infants and young children for our population, exposed to PFOA mainly via drinking water, might be explained by a number of factors, including *in utero* exposure, high uptake by the child during breast-feeding, a relatively higher bioconcentration by the infant perhaps related to lower excretion, and a higher rate of water consumption relative to body size for children than for mothers.

Children in this study were all at least 1 year of age at the time of the survey, so we have no measure of cord blood or early postnatal levels as a direct measure of *in utero* exposure. However, a number of published studies suggest the magnitude of *in utero* exposure by measuring cord and maternal PFAA concentrations. The overall cord:maternal ratio for paired samples in different populations ranged from 0.67 to 0.87 for PFOA and from 0.28 to 0.56 for PFOS (Fei et al. 2007; Fromme et al. 2010; Hanssen et al. 2010; Inoue et al. 2004; Kim et al. 2011; Monroy et al. 2008; Needham et al. 2011).

The higher ratios for PFOA (1.83) and PFOS (1.35) for children from mothers intending to breast-feed exclusively than for those intending to breast- and/or bottle feed (1.14 for PFOA and 1.12 for PFOS) suggest that children could be more exposed via maternal milk. Important limitations to this finding, however, are that breast-feeding information was available only for 35 matched pairs and that breast-feeding was measured as the mother’s stated intent at the time of delivery rather than whether she actually breast-fed. Additionally, we have no information on duration of breast-feeding or whether bottle-fed babies received formula made with PFOA-contaminated water. These results, however, are consistent with a recently published study in which breast milk was shown to contribute more than 94% and 83% of the total PFOS and PFOA exposure, respectively, in infants 6 months of age, despite the low PFAA concentrations in breast milk (Haug et al. 2011).

Considering that PFOA in the body is subject to excretion [with half-lives between 2.3 and 3.8 years reported for adults (Bartell et al. 2010; Olsen et al. 2007)] and the dilution due to rapid growth and increasing body weight at young ages, the *in utero* exposure and absorption from lactation are not enough to explain the elevated PFOA concentrations in children < 12 years of age. For a child and mother having similar potential exposure (living in the same home with shared water supply), on average the child has a serum PFOA concentration around 20–30% higher than that of the mother. This might suggest that there is increased bioconcentration of the chemical during these young years. The excess PFOA in children compared with their mothers is higher in districts where PFOA contamination is higher than in less-contaminated districts where water forms a lower proportion of the daily intake of PFOA. Whether the excess in children reflects higher average water intake or lower excretion rate cannot be ascertained from this study. By the age of 12 years, it appears that the levels of water intake and half-life (if that varies with age) have converged so that, on average, child and mother PFOA serum concentrations are similar.

The patterns for PFOS are quite different, with the elevated child:mother ratio hardly varying with the child’s age. This might be explained by the type of exposure to PFOS being from different types of sources between the mother and child, and such differences persist throughout childhood, although presumably changing with age. This persistent difference in mother and child serum PFOS concentrations raises interesting questions about their respective intakes, and addressing these questions would help us to understand population exposure to these chemicals. For PFOA, where the exposure is largely via drinking water, it appears that by 12 years of age, children’s and adults’ intake patterns and metabolisms become similar.

## Conclusions

We observed that children had higher PFOA concentrations than did their mothers. The ratio was the highest among children ≤ 5 years of age; on average, these children had PFOA serum concentrations 44% higher than their mothers. The ratio was significantly higher for boys than for girls at > 5 years of age. In a population exposed to elevated PFOA concentrations via contaminated drinking water, children seemed to concentrate the chemical more than their mothers up to about age 12 years. This is probably attributable to exposure via drinking water as well as exposure *in utero* and via breast milk. Children had higher PFOS concentrations than did their mothers, and this persisted at least until 19 years of age, with concentrations in children on average 42% higher than in their mothers. *In utero* and lactational exposure appears to make less of a contribution for PFOS than for PFOA. Further studies are warranted on the mother–child PFAA relationship to understand how children’s exposure and rate of uptake vary as children grow.

## Supplemental Material

(90 KB) PDF
