# Supplementary material for: Relationships of Perfluorooctanoate and Perfluorooctane Sulfonate Serum Concentrations between Mother–Child Pairs in a Population with Perfluorooctanoate Exposure from Drinking Water
Source: Environ Health Perspect. 2012 Jan 23;120(5):752–7. doi: 10.1289/ehp.1104538 (PMC3346790; doi:10.1289/ehp.1104538)
Supplement: (90 KB) PDF [file ehp.1104538.s001.508.pdf]

Supplemental Material

Relationships of Perflurooctanoate and Perfluorooctane Sulfonate Serum Concentrations Between Child-Mother Pairs in a Population with Perflurooctanoate Exposure from Drinking Water

Debapriya Mondal, Maria-Jose Lopez-Espinosa, Ben Armstrong, Cheryl R. Stein, Tony Fletcher

Table of contents

1. Supplemental Material, Table 1 Page 2
2. Supplemental Material, Table 2 Page 3
3. Supplemental Material, Table 3 Page 4
4. Supplemental Material, Table 4 Page 5
5. Supplemental Material, Table 5 Page 6
6. Supplemental Material, Table 6 Page 7
7. Supplemental Material, Table 7 Page 8
8. References Page 9

Supplemental Material, Table 1. Classification of matched child-mother pairs, Mid-Ohio Valley, 2005-2006

Classification	Criteria
Perfect	Child's month/year of birth and gender match in "child and mother files" and mother's first/last name is recorded as the parent in the "guardian file".
Excellent	Child's month/year of birth, gender and last names match in "child and mother files" but the mother cannot be confirmed in the "guardian file".
Good	Child's month/year of birth and gender match in "child and mother files" but last names do not match and the mother cannot be confirmed in the "guardian file" OR last names or address and gender match in "child and mother files" but child's month/year of birth do not match in the "child and mother files" but mother's first/last name is recorded as the parent in the "guardian file".
Probable	Last name or address and gender match in "child and mother files" but child's month/year of birth do not match in the "child and mother files" and the mother cannot be confirmed in the "guardian file" OR mother's first/last name is recorded as the parent in the "guardian file" but child's month/year of birth and last names or address do not match in the "child and mother files".

Supplemental Material, Table 2. Detail of frequency of successful child-mother matches by age and type of match, Mid-Ohio Valley, 2005-2006

Child age	Total (Children)	Matched	% of matched children	Type of match[a]			
				perfect	excellent	good	probable
1	41	31	75.61	25	2	5	0
2	195	162	83.08	136	13	13	0
3	274	225	82.12	180	20	22	3
4	329	267	81.16	213	27	25	2
5	326	266	81.60	214	27	24	1
6	362	284	78.45	242	22	19	1
7	393	317	80.66	252	39	24	2
8	417	321	76.98	265	31	23	2
9	464	368	79.31	308	27	31	2
10	507	389	76.73	304	38	45	2
11	482	295	61.20	180	25	78	12
12	522	386	73.95	314	38	32	2
13	566	424	74.91	324	55	41	4
14	608	458	75.33	381	38	33	6
15	676	491	72.63	373	74	41	3
16	684	444	64.91	348	53	39	4
17	656	439	66.92	332	67	37	3
18	685	374	54.60	42	267	54	11
19	705	359	50.92	8	285	54	12
Total	8,893	6,301	70.85	4,441	1,148	640	72

[a]Defined in Supplemental Material, Table 1

Supplemental Material, Table 3. Overall Spearman correlation between child and mother PFAAs by type of match

Variable	Match type[a]	*n*	rho
PFOA	all	5,572	0.81
	perfect	3,872	0.82
	excellent	1,071	0.80
	good	546	0.78
	probable	63	0.66
PFOS	all	5,572	0.27
	perfect	3,872	0.27
	excellent	1,071	0.27
	good	546	0.31
	probable	63	0.43

[a]Defined in Supplemental Material, Table 1
PFAA, perfluoroalkyl acid; PFOA, perfluorooctanoate; PFOS, perflurooctane sulfonate.

Supplemental Material, Table 4. Results of regression analyses of child’s serum PFOA and PFOS concentration (y) on mother’s (x)

			All			Stable[a]		
Contaminant	Child's age	Constraint	Intercept (95% CI)	Slope (95% CI)	R^2	Intercept (95% CI)	Slope (95% CI)	R^2
PFOA	All (1-19)	No	0.91 (0.86, 0.96)	0.76 (0.75, 0.79)	0.695	0.78 (0.70, 0.87)	0.81 (0.79, 0.83)	0.736
		Yes	0.14 (0.12, 0.16)	1		0.10 (0.07, 0.13)	1	
	≤ 5	No	0.89 (0.72, 1.06)	0.84 (0.79, 0.89)	0.701	0.64 (0.40, 0.88)	0.91 (0.84, 0.97)	0.741
		Yes	0.36 (0.30, 0.43)	1		0.32 (0.24, 0.39)	1	
	6 -10	No	0.93 (0.83, 1.02)	0.8 (0.77, 0.83)	0.724	0.88 (0.73, 1.03)	0.82 (0.78, 0.86)	0.744
		Yes	0.27 (0.23, 0.30)	1		0.23 (0.18, 0.29)	1	
	> 10	No	0.90 (0.84, 0.96)	0.74 (0.72, 0.76)	0.696	0.71 (0.60, 0.83)	0.80 (0.77, 0.83)	0.753
		Yes	0.04 (0.02, 0.07)	1		−0.04 (−0.08, 0.01)	1	
PFOS	All (1-19)	No	2.31 (2.25, 2.37)	0.25 (0.22, 0.27)	0.088	2.28 (2.17, 2.39)	0.26 (0.22, 0.29)	0.085
		Yes	0.36 (0.33, 0.38)	1		0.32 (0.29, 0.35)	1	
	≤ 5	No	2.21 (2.02, 2.39)	0.23 (0.16, 0.30)	0.073	2.15 (1.88, 2.41)	0.25 (0.15, 0.35)	0.082
		Yes	0.29 (0.22, 0.36)	1		0.23 (0.14, 0.33)	1	
	6 -10	No	2.51 (2.39, 2.63)	0.22 (0.17, 0.26)	0.064	2.41 (2.18, 2.63)	0.25 (0.17, 0.34)	0.063
		Yes	0.49 (0.45, 0.53)	1		0.46 (0.40, 0.52)	1	
	> 10	No	2.25 (2.18, 2.33)	0.26 (0.23, 0.28)	0.101	2.25 (2.12, 2.39)	0.26 (0.21, 0.30)	0.104
		Yes	0.31 (0.28, 0.33)	1		0.27 (0.22, 0.31)	1	

[a]Stable: the mother and child remained in the same water district (the six contaminated districts) from the child birth up to the survey. PFAA, perfluoroalkyl acid; PFOA, perfluorooctanoate; PFOS, perflurooctane sulfonate.

Two regression analyses of logged child’s on mother’s PFOA and PFOS are shown for each age group as a) an unconstrained linear regression and b) a regression constraining the slope to be equal to one.

The unconstrained regression consistently shows slopes less than one. This pattern is, however, expected even where true slopes are equal to one if the x as well as the y variable is subject to error, such as anticipated here due to short-term within-person variation (Armstrong. 1998). This reasoning, together with advantages in simplifying the relationship, motivates the regression constrained with slope constrained to equal one, which is equivalent to estimating the ratio of geometric means. (exp(intercept)=GM(child)/GM(mother)).

Supplemental Material, Table 5. Geometric mean of mother and child PFOA concentrations and their ratio and Spearman correlation coefficients by age of child, Mid-Ohio Valley, 2005-2006

Child age (years)	*n*	GM PFOA (ng/mL)		child: mother GM ratio	rho
		mother	child		
2	59	23.17	33.71	1.45	0.74
3	117	28.55	42.75	1.50	0.86
4	147	25.12	38.00	1.51	0.82
5	162	27.94	37.27	1.33	0.81
6	194	28.44	39.13	1.38	0.83
7	248	26.13	37.14	1.42	0.87
8	259	26.64	34.25	1.29	0.84
9	313	28.00	35.99	1.29	0.86
10	320	27.23	33.67	1.24	0.83
11	194	31.05	34.44	1.11	0.84
12	340	29.60	33.16	1.12	0.85
13	372	26.00	26.79	1.03	0.82
14	406	24.66	25.44	1.03	0.84
15	435	26.09	27.97	1.07	0.83
16	390	28.67	28.57	1.00	0.81
17	391	26.51	26.94	1.02	0.81
18	306	28.45	30.60	1.08	0.78
19	290	27.85	27.85	1.00	0.76

GM, geometric mean ; PFOA, perfluorooctanoate.

Supplemental Material, Table 6. Geometric mean of mother and child PFOS concentrations and their ratio and Spearman correlation coefficient by age of child, Mid-Ohio Valley, 2005-2006

Child age (years)	*n*	GM PFOS (ng/mL)		child: mother GM ratio	rho
		mother	child		
2	59	11.92	11.73	0.98	0.30
3	117	11.76	15.88	1.35	0.16
4	147	12.16	15.68	1.29	0.26
5	162	11.96	18.49	1.55	0.36
6	194	12.86	19.43	1.51	0.26
7	248	12.90	20.55	1.59	0.13
8	259	12.96	22.27	1.72	0.28
9	313	13.63	23.45	1.72	0.27
10	320	13.67	21.66	1.58	0.28
11	194	13.38	20.72	1.55	0.19
12	340	13.69	19.61	1.43	0.33
13	372	13.95	18.58	1.33	0.36
14	406	12.92	17.66	1.37	0.23
15	435	13.86	18.52	1.34	0.25
16	390	14.28	19.37	1.36	0.24
17	391	13.48	18.44	1.37	0.31
18	306	14.23	19.00	1.34	0.37
19	290	13.75	17.49	1.27	0.17

GM, geometric mean; PFOS, perflurooctane sulfonate

Supplemental Material, Table 7. Geometric means and ranges (ng/mL) of maternal PFOA for the matched child-mother pairs in three exposure groups, Mid-Ohio Valley, 2005-2006

Exposure[a]	WD	N	GM	P 10th, P 90th
High	3	416	210.2	75.6, 601.7
Medium	1,2,4	875	33.7	14.2, 89.8
Low	5,6	472	10.3	4.9, 22.9

GM, geometric mean; P, percentile; PFOA, perfluorooctanoate; WD, water district;
[a] the mother and child remained in the same water district (the six contaminated districts) from the child birth up to the survey

References

Armstrong BG. 1998. Effect of measurement error on epidemiological studies of environmental and occupational exposures. Occup Environ Med 55: 651-656.
